# Characterization of Putative *cis*-Regulatory Elements in Genes Preferentially Expressed in *Arabidopsis* Male Meiocytes

**DOI:** 10.1155/2014/708364

**Published:** 2014-08-27

**Authors:** Junhua Li, Jinhong Yuan, Mingjun Li

**Affiliations:** ^1^College of Life Sciences, Henan Normal University, Xinxiang, Henan 453007, China; ^2^Institute of Vegetables and Flowers, Chinese Academy of Agricultural Sciences, Beijing 100081, China

## Abstract

Meiosis is essential for plant reproduction because it is the process during which homologous chromosome pairing, synapsis, and meiotic recombination occur. The meiotic transcriptome is difficult to investigate because of the size of meiocytes and the confines of anther lobes. The recent development of isolation techniques has enabled the characterization of transcriptional profiles in male meiocytes of *Arabidopsis*. Gene expression in male meiocytes shows unique features. The direct interaction of transcription factors (TFs) with DNA regulatory sequences forms the basis for the specificity of transcriptional regulation. Here, we identified putative *cis*-regulatory elements (CREs) associated with male meiocyte-expressed genes using *in silico* tools. The upstream regions (1 kb) of the top 50 genes preferentially expressed in *Arabidopsis* meiocytes possessed conserved motifs. These motifs are putative binding sites of TFs, some of which share common functions, such as roles in cell division. In combination with cell-type-specific analysis, our findings could be a substantial aid for the identification and experimental verification of the protein-DNA interactions for the specific TFs that drive gene expression in meiocytes.

## 1. Introduction

Meiosis is a special type of cell division that, after two consecutive rounds of nuclear divisions, leads to the production of haploid gametes. The processes of homologous chromosome pairing, synapsis, and meiotic recombination all occur during meiosis. Meiotic recombination is essential for plant reproduction and breeding because it ensures equal segregation and genetic exchange between homologous chromosomes [1–4]. The male meiocytes of* Arabidopsis* occupy only a small fraction of the anther tissue and are surrounded by somatic anther lobes [5]. An effective meiocyte collection method was established only recently; this development has enabled investigations of the meiotic transcriptome [5, 6]. Genome-wide gene expression analysis revealed unique transcriptome landscapes during male meiosis [5, 6].

Gene expression in eukaryotic cells is regulated by transcription factors (TFs). There are around 2000 TFs in the* Arabidopsis *genome [7], and interactions of the DNA-binding domains of TFs with specific* cis*-regulatory elements (CREs) can activate the expression of several to many thousands of target genes. The transcriptional domains of regulatory genes are critically important in many developmental processes [8]. Meiosis operates in a highly specified cell cluster and thus requires precise spatial and temporal control [3]. In* Arabidopsis*, the expression of many meiotic genes such as* AtDMC1* [9, 10],* SDS* [11],* MMD1* [12], and* RCK *[13] is highly regulated. Studying the commonness and distribution of CREs in the promoters of coexpressed genes can help facilitate the identification of signaling networks in specific cell types (e.g., [14–17]). For example, CREs or promoter motifs have been investigated in sperm cells (mature pollen) of both rice and* Arabidopsis* [18, 19].

Transcriptome profiling experiments have shown that more than 1,000 genes were preferentially expressed in meiocytes [5]; a high proportion of the promoters of such preferentially expressed genes were sufficient to drive green fluorescent protein (GFP) reporter activity in meiocytes [20]. These preliminary studies laid a substantial foundation that has enabled the mining and the examination of the common structures of meiotically active promoters. In this study, the sequences of 50 meiotically active promoters were analyzed. The putative CREs in these promoters were identified; these CREs may be responsible for the high activity of these promoters in male meiocytes.

## 2. Materials and Methods

We selected candidate genes from data generated in a previous mRNA deep-sequencing study of meiosis-specific genes in* Arabidopsis *[5]. These included the most highly expressed genes in male meiocytes. In a list with genes that had ≥4 times higher expression in meiocytes than in anthers, top 50 genes in the meiocytes to seedling comparison list were chosen with exclusion of transposable element genes. The difference in expression between meiocytes and anther, the difference in expression between meiocytes and seedlings, the annotated function, and the GO (gene ontology) functional categorization of the 50 top genes are presented in Supplemental File 1, available online at http://dx.doi.org/10.1155/2014/708364. As a negative control, 50 genes randomly selected from an Affymetrix ATH1 microarray experiment deposited in the NASC database were analyzed [21]; see Supplemental File 2 for descriptions of these control genes.

One Kb of upstream sequences relative to the transcription start sites were retrieved using Regulatory Sequence Analysis Tools (RSAT, http://rsat.ulb.ac.be/rsat/) [22]. Analysis of known CREs was initially performed using SIGNALSCAN program in plant* cis*-acting regulatory DNA elements (PLACE, http://www.dna.affrc.go.jp/PLACE/) [23, 24]. Analysis of statistically overrepresented elements was conducted by Pscan (http://159.149.160.51/pscan/) [25]. In the Pscan window, TAIR gene identifiers of the 50 genes were submitted, the source organism was specified as* Arabidopsis thaliana*, and the region to be analyzed was from −1000 to +0 with regard to the annotated transcription start site. For assessing the significance of the results, the *P* values were computed by Pscan with a *z*-test, a test that associated with each profile the probability of obtaining the same score on a random sequence set [25]. An element is considered to be significantly overrepresented if the *P* value is less than 0.01. Additional analysis for unknown novel motifs was conducted by Promzea (http://promzea.org) [26]. 1000 bp long promoter regions were analyzed and each predicted motif was provided with a mean normalized conditional probability (MNCP); a MNCP score greater than 1 indicates that the motif is more represented in the input data set compared to a random set of promoters/first introns [26]. Motifs predicted by Promzea were compared with experimentally defined motifs in the PLACE database using STAMP [27]. Strand bias analysis of putative CREs was performed using Athamap (http://www.athamap.de/) [28–32], −1000 to 0 regions relative to the transcription start site were analyzed, and the total strand distribution of CREs was the sum of the individual CRE numbers in each promoter in the “overview” search result.

## 3. Results and Discussions

**Figure 1:**
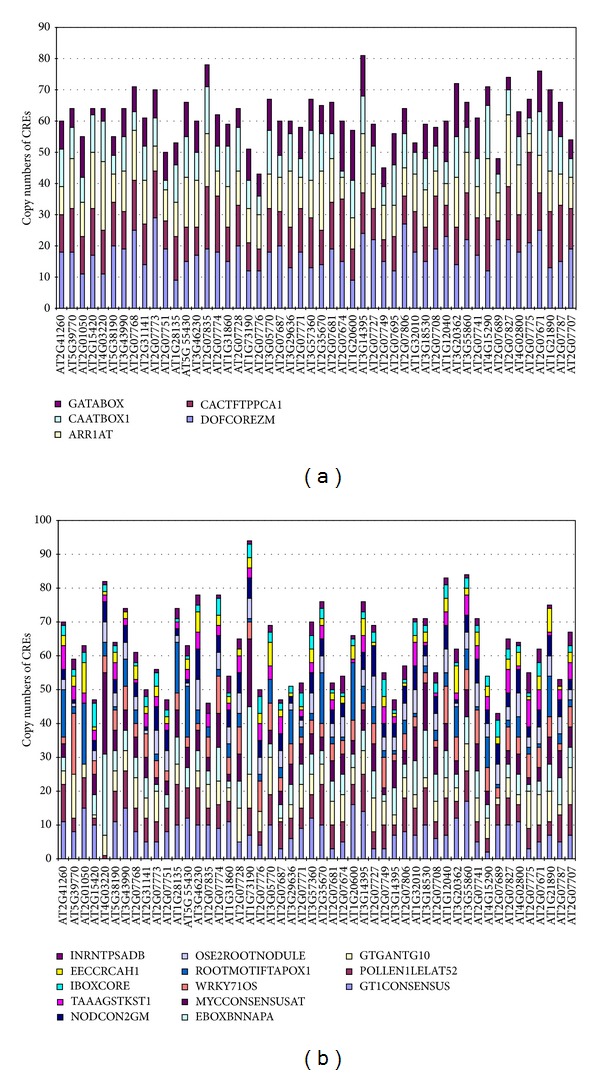
Distribution and occurrence of enriched PLACE motifs in the promoters of 50 genes preferentially expressed during meiosis. (a) Five common CREs found in all of the 50 promoters; (b) 13 CREs present in at least 80% of the promoters.

Putative 1000 bp promoter regions were selected and their CREs were analyzed by the use of the PLACE collection. Five CREs were found in all 50 promoters: DOFCOREZM (5′-AAAG-3′), CACTFTPPCA1 (5′-YACT-3′, Y=T/C), ARR1AT (5′-NGATT-3′, N=G/A/C/T), CAATBOX1 (5′-CAAT-3′), and GATABOX (5′-GATA-3′). The frequencies and distributions of these CREs in each promoter are shown in Figure 1(a).

DOFCOREZM was the most abundant CRE in the 50 putative promoter sequences. It is a core site for the binding of Dof proteins in maize. The Dof proteins are a family of plant-specific TFs that includes Dof1, Dof2, Dof3, and PBF [33, 34]. Maize Dof1 was suggested to be a regulator of the expression of the C4 photosynthetic phosphoenolpyruvate carboxylase (*C4PEPC*) gene [35]. Dof1 also enhances transcription of the cytosolic orthophosphate dikinase (*cyPPDK*) genes and the nonphotosynthetic* PEPC* gene [33]. Maize Dof2 suppresses the promoter of* C4PEPC* [35]; PBF is an endosperm-specific Dof protein that binds to the prolamin box of a native B-hordein promoter in barley endosperm [36]. CACTFTPPCA1 is a key component of* Mem1* (*mesophyll expression module 1*) and is found in the distal promoter region of the C4 isoform of phosphoenolpyruvate carboxylase (*ppcA1*) in the C4 dicot* Flaveria trinervia*; it determines the mesophyll-specific expression of* ppcA1* [37]. ARR1AT is the binding element of ARR1 found in* Arabidopsis*. ARR1 is a response regulator [38]. CAATBOX1 is responsible for the tissue specific promoter activity of a pea legumin gene [39]. GATABOX is required for light-dependent and nitrate-dependent control of transcription in plants [40]. The GATA motif has been found in the promoter ofthe* Cab22* gene that encodes the* Petunia* chlorophyll a/b binding protein; this motif is the specific binding site of ASF-2 [41].

In addition to the five CREs that were found in all 50 promoters, there are 13 CREs that were found in at least 80% of the promoters (Figure 1(b)). These include GT1CONSENSUS (5′-GRWAAW-3′, R=A/G, W=A/T), POLLEN1LELAT52 (5′-AGAAA-3′), GTGANTG10 (5′-GTGA-3′), EBOXBNNAPA (5′-CANNTG-3′, N=G/A/C/T), MYCCONSENSUSAT (5′-CANNTG-3′, N=G/A/C/T), WRKY71OS (5′-TGAC-3′), ROOTMOTIFTAPOX1 (5′-ATATT-3′), OSE2ROOTNODULE (5′-CTCTT-3′), NODCON2GM (5′-CTCTT-3′), TAAAGSTKST1 (5′-TAAAG-3′), IBOXCORE (5′-GATAA-3′), EECCRCAH1 (5′-GANTTNC-3′, N=G/A/C/T), and INRNTPSADB (5′-YTCANTYY-3′, Y=T/C, N=G/A/C/T). Among these, seven elements are found in genes specifically expressed in particular organ. POLLEN1LELAT52 is one of two codependent regulatory elements responsible for pollen specific activation of tomato (*Lycopersicon esculentum*)* LAT52* gene [42]. GTGANTG10 is found in the promoter of the tobacco late pollen gene* g10* [43]. EBOXBNNAPA is a motif associated with storage proteins [44]. TAAAGSTKST1 is a target site in the control of guard cell-specific gene expression [45].

Six of the 13 CREs distributed in at least 80% of the examined promoters are annotated as being involved in plant responses to environmental factors, for example, GT1CONSENSUS for light and salicylic acid [46, 47], MYCCONSENSUSAT for cold [48–50], WRKY71OS for gibberellin and pathogenesis [51, 52], IBOXCORE and INRNTPSADB for light [53–55], and EECCRCAH1 for CO_2_ [56, 57]. The involvement of these CREs in responses to environmental factors points to possible roles for these elements in combining signals from meiotic process and environmental factors, especially light and stress.

The aforementioned PLACE motifs represent the basic CREs required for a promoter but may not be statistically overrepresented as compared with the average level of CREs in the* Arabidopsis *genome. Among the PLACE motifs that were present in at least 80% of the promoters we examined (Figure 1), 17 of 18 are also present in rice sperm cell-specific genes [18]. The only exception to this striking similarity was the EECCRCAH1 CRE.

**Figure 2:**
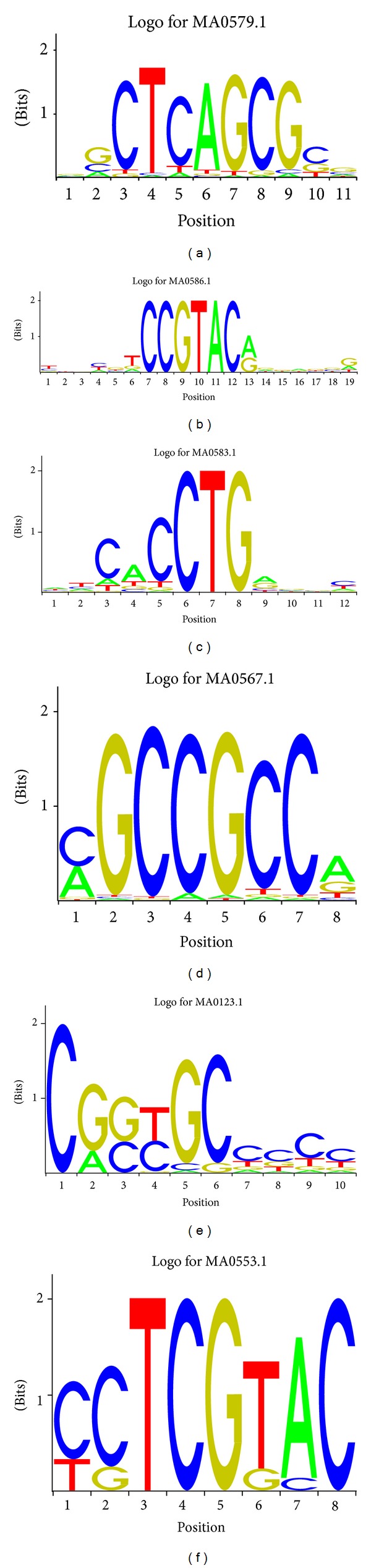
Sequence logos of overrepresented sequences in the promoters of genes preferentially expressed during meiosis, detected using Pscan. Letters in the logos abbreviate the nucleotides (A, C, G, and T) and are sized relative to their occurrence.

We further searched for motifs that were statistically overrepresented. That is, the frequency of an element in the 50 examined promoters is above the average level of the* Arabidopsis* genome. Six overrepresented putative TF binding site motifs were identified in our Pscan analysis (Figure 2 and Table 1). When we used 50 randomly selected genes (negative control) as input, only one such overrepresented motif was detected (Supplemental File 3), indicating that the meiotically active promoter sequences possess more conserved sequences.

The most significantly abundant motif detected by Pscan, CTCAGCG, is the binding sequence of* Arabidopsis* CELL DIVISION CYCLE 5 (AtCDC5), which is expressed extensively in shoot and root meristems and may function in cell cycle regulation [58, 59]. This result suggests that similar regulatory machinery functions in meiocytes and meristems and that such machinery leads to high mitotic or meiotic cell division activity. Another overrepresented motif contains the core binding motif GTAC that is recognized by the plant-specific SQUAMOSA promoter binding protein (SBP) domain transcription factor AtSPL14, which is involved in plant development and resistance to programmed cell death [60]. The binding motif of the RAV1 (RAV: for related to ABI3/VP1) DNA binding protein is overrepresented in the Pscan search results [61]; RAV1 is a regulator of plant development and is involved in plant responses to biotic and abiotic stress [62–65]. Another overrepresented motif is recognized by ERF1; a TF that belongs to the EREB/AP2 family and regulates plant responses to jasmonate, ethylene, and fungi [66–70]. The statistically overrepresented CE-1 like sequence CACCG is an ABA response sequence in a number of ABA-related genes, and it is the target of the maize abscisic acid insensitive 4 (ABI4) protein [71]. Another Pscan motif, the SCHLAFMÜTZE (SMZ) binding site, is the target of an AP2-like transcription factor that acts as a repressor of flowering [72].

**Figure 3:**
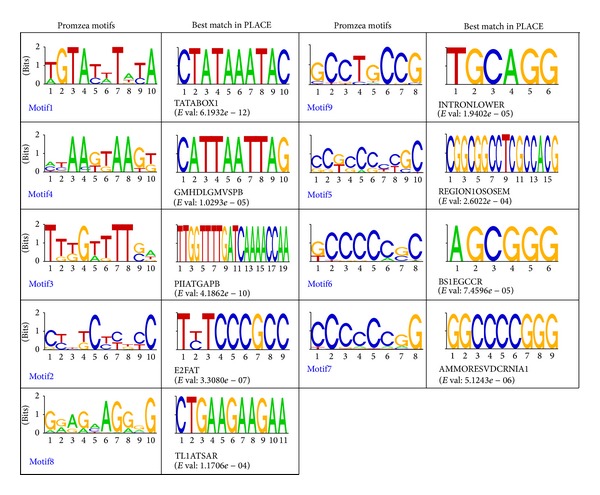
Sequence logos of novel motifs in the promoters of genes preferentially expressed during meiosis, detected using Promzea. The best match of each motif in the PLACE database is indicated in the panels to the right. Letters in the logos abbreviate the nucleotides (A, C, G, and T) and are sized relative to their occurrence. The *e*-value for STAMP is indicated by the false discovery ratio (FDR).

As a complement to our Pscan analysis, we searched for novel promoter DNA motifs associated with upregulation in* Arabidopsis* meiocytes using the Promzea motif discovery tool [26]. Nine overrepresented motifs were detected by Promzea with MNCP scores >1 in the promoters of the 50 meiotically active genes; five were detected in the promoters of the 50 randomly selected control genes (Supplemental File 4). This result supports the result from the Pscan analysis that meiotically active promoters possess more conserved motifs than randomly selected promoters. The 14 motifs matched to different experimentally defined motifs in the literature (Figure 3 and Supplemental File 5).

Motif1 from the Promzea analysis was statistically close to the TATABOX1 element, an element that is critical for the initiation of tissue specific transcription (Figure 3) [73, 74]. Motif4 matched the phosphate response domain GMHDLGMVSPB [75]. Motif3 matched the experimentally defined motif PIIATGAPB, which is responsible for light-activated gene expression [75]. Motif2 matched the E2FAT motif that is the binding site of E2F. The E2F transcription factors control the cell cycle by regulating the transcription of genes required for cell cycle and DNA replication [76]; these processes are obviously important in meiosis. Motif8 was similar to the pathogen/elicitor-related element TL1ATSAR [77]. Of the nine motifs predicted by Promzea, four motifs (Motif5, Motif6, Motif7, and Motif9) were enriched with CG, a property found in regulatory elements that is related to DNA methylation. CpG methylation is known to suppress transcription [78]. The presence of CG-enriched motifs identified in our analysis suggests that like gene activation, gene repression is also important for meiotically active gene regulatory networks, for example, the suppression of meiosis-restricted processes in somatic tissues. In addition, motif comparison analysis using STAMP found that these motifs possess other properties: Motif9 matched to INTRONLOER that is involved in 3′ intron-exon splice junctions in plants [79], Motif5 matched to REGION1OSOSEM that is involved in the control of transcription by ABA [80], Motif6 matched to the tissue specific expression element BS1EGCCR [81], and Motif7 matched to the ammonium response element AMMORESVDCRNIA1 [82].

In the promoters of the negative genes, CREs are almost equally distributed on both the sense and the antisense strands (CREs on sense strand/CREs on antisense strand = 2742/2706 = 1/0.987); however, comparatively large numbers of CREs are located on the antisense strand compared to the sense strand of the meiotically active promoters (CREs on sense strand/CREs on antisense strand = 2758/2941 = 1/1.066). Interestingly, a similar bias of CRE distribution on the antisense strand is observed in promoters of rice sperm cell-specific genes [18].

The information from this study can be used in efforts to characterize the interactions between regulatory elements and TFs in meiocytes. Cell-type-specific analysis of TF expression is one of the strategies for sorting true protein-DNA interaction from numerous potentially spurious candidates [83]. For example, one of the PLACE motifs identified in this study, the GATABOX (Figure 1(a)), is the binding motif of the conserved C2C2-GATA TFs that have two GATA zinc fingers [40]. There are 29 C2C2-GATA family members that have been identified in* Arabidopsis*. They are highly expressed in early flower domains, and a few are involved in flower development [84, 85]. In our analysis, we identified two members of this family of TFs that are highly expressed in male meiocytes (*AT5G47140* and* AT1G08000*, Table 2). Therefore, AT5G47140 and AT1G08000 are better candidates than other C2C2-GATA family members for being proteins that can bind to GATABOX CREs in meiocytes. E2F transcription factors are essential for the regulation of the cell cycle and DNA replication. Three classical E2F proteins (E2Fa–c) and three atypical E2F proteins (E2Fd–f) have been characterized in* Arabidopsis *[86, 87]. Among these,* E2Fa* (*AT2G36010*) and* E2Fe* (*AT3G48160*) are highly expressed in meiocytes (Table 2); they may therefore be better candidates than other E2Fs for being proteins that can bind to E2FAT-like CREs in meiocytes, and this may link the E2Fs to the control of meiotic processes such as the meiotic cell cycle and DNA replication.

More than half of the overrepresented CREs identified in this study are binding sites of TFs that function in plant responses to environmental factors. We therefore infer that, during meiosis, exogenous signals are perceived largely through particular CRE and that this is especially likely for light and stress signals [89–92].

## 4. Conclusions

In this study, which aimed to identify CREs associated with genes preferentially expressed during meiosis, we analyzed 1 kb upstream regions of the 50 genes that were highly expressed in* Arabidopsis* meiocytes. Although the CREs in 10 promoters of meiotically active genes were analyzed in our previous study [20], here we performed a more comprehensive* in silico* study with a larger number of genes. The CREs that we identified in the promoters of these 50 genes may be responsible for the high activity of corresponding promoters in male meiocytes. The information obtained from this study can be used to identify TFs that regulate meiotically active gene expression and, more attractively, the synthesis of artificial promoters that could drive high gene expression in meiocytes. As meiosis is evolutionarily conserved, the information on transcriptional domains obtained from the model system* Arabidopsis* has value not only in assessing the conservation of functional pathways in meiosis of other eukaryotes but also in applications seeking to improve crop plants.

## Supplementary Material

Supplemental File 1 presents the difference in expression between meiocytes and anther, the difference in expression between meiocytes and seedlings, the annotated function, and the GO functional categorization of the 50 candidate genes used for promoter study. Supplemental File 2 lists the 50 randomly selected genes used as a negative control. Supplemental File 3 shows the descriptions of the overrepresented sequence in the promoters of 50 randomly selected genes, detected using Pscan. Supplemental File 4 shows the sequence logos of novel motifs in the promoters of 50 genes preferentially expressed during meiosis and 50 randomly selected genes, detected using Promzea. Supplemental File 5 shows the sequence logos of novel motifs in the promoters of 50 randomly selected genes, detected using Promzea, and the best match of each motif in the PLACE database.

## Figures and Tables

**Table 1:** Description of the most abundant motifs in promoters of genes preferentially expressed during meiosis, detected using Pscan.

Pscan ID	TF name	Class of the TF	Family of the TF	*P* value	References
MA0579.1	CDC5	Helix-turn-helix	Myb	1.89231*e* − 05	Hirayama and Shinozaki [58]
MA0586.1	SPL14	Zinc-coordinating	SBP	6.00715*e* − 05	Liang et al. [60]
MA0583.1	RAV1	EcoRII-fold	ABI3VP1	0.000477606	Kagaya et al. [61]
MA0567.1	ERF1	Beta-Hairpin-Ribbon	AP2 MBD-like	0.000997537	Godoy et al. [88]
MA0123.1	ABI4	Beta-Hairpin-Ribbon	AP2 MBD-like	0.00108575	Niu et al. [71]
MA0553.1	SMZ	AP2-ERF	AP2-ERF	0.00224012	Unpublished

**Table 2:** Transcriptional factor genes preferentially expressed during meiosis with putative target binding sites highly enriched in meiocytes. Numbers in the boxes are ratios of read counts that indicate the difference in expression in bidirectional comparisons between each of the tissue pairs. M: meiocytes; A: anther; S: seedling.

Gene ID (name)	M/A	M/S	A/S
*AT5G47140 *	2.30	4.09	1.78
*AT1G08000 *	1.02	2.43	2.37
*AT2G36010 *(*E2Fa*)	0.73	2.19	3.01
*AT3G48160 *(*E2Fe*)	0.76	2.19	2.88

## References

[1] Yanowitz J (2010). Meiosis: making a break for it. *Current Opinion in Cell Biology*.

[2] Ma H (2006). A molecular portrait of Arabidopsis meiosis. *The Arabidopsis Book*.

[3] Ma H (2005). Molecular genetic analyses of microsporogenesis and microgametogenesis in flowering plants. *Annual Review of Plant Biology*.

[4] Ronceret A, Sheehan M, Pawlowski W (2008). Chromosome dynamics in meiosis. *Cell Division Control in Plants*.

[5] Chen C, Farmer AD, Langley RJ (2010). Meiosis-specific gene discovery in plants: RNA-Seq applied to isolated Arabidopsis male meiocytes. *BMC Plant Biology*.

[6] Yang H, Lu P, Wang Y, Ma H (2011). The transcriptome landscape of Arabidopsis male meiocytes from high-throughput sequencing: the complexity and evolution of the meiotic process. *Plant Journal*.

[7] Guo A, He K, Liu D (2005). DATF: a database of *Arabidopsis* transcription factors. *Bioinformatics*.

[8] Watanabe K, Okada K (2003). Two discrete *cis* elements control the abaxial side-specific expression of the *FILAMENTOUS FLOWER* gene in *Arabidopsis*. *Plant Cell*.

[9] Klimyuk VI, Jones JDG (1997). *AtDMC1*, the *Arabidopsis* homologue of the yeast *DMC1* gene: characterization, transposon-induced allelic variation and meiosis-associated expression. *Plant Journal*.

[10] Couteau F, Belzile F, Horlow C, Grandjean O, Vezon D, Doutriaux M (1999). Random chromosome segregation without meiotic arrest in both male and female meiocytes of a *dmc1* mutant of *Arabidopsis*. *Plant Cell*.

[11] Azumi Y, Liu D, Zhao D (2002). Homolog interaction during meiotic prophase I in *Arabidopsis* requires the *SOLO DANCERS* gene encoding a novel cyclin-like protein. *EMBO Journal*.

[12] Yang X, Makaroff CA, Ma H (2003). The Arabidopsis *MALE MEIOCYTE DEATH1* gene encodes a PHD-finger protein that is required for male meiosis. *Plant Cell*.

[13] Chen C, Zhang W, Timofejeva L, Gerardin Y, Ma H (2005). The Arabidopsis *ROCK-N-ROLLERS* gene encodes a homolog of the yeast ATP-dependent DNA helicase MER3 and is required for normal meiotic crossover formation. *Plant Journal*.

[14] Tullai JW, Schaffer ME, Mullenbrock S, Kasif S, Cooper GM (2004). Identification of transcription factor binding sites upstream of human genes regulated by the phosphatidylinositol 3-kinase and MEK/ERK signaling pathways. *The Journal of Biological Chemistry*.

[15] DeRisi JL, Iyer VR, Brown PO (1997). Exploring the metabolic and genetic control of gene expression on a genomic scale. *Science*.

[16] Harmer SL, Hogenesch JB, Straume M (2000). Orchestrated transcription of key pathways in Arabidopsis by the circadian clock. *Science*.

[17] Nemhauser JL, Mockler TC, Chory J (2004). Interdependency of brassinosteroid and auxin signaling in Arabidopsis. *PLoS Biology*.

[18] Sharma N, Russell SD, Bhalla PL, Singh MB (2011). Putative cis-regulatory elements in genes highly expressed in rice sperm cells. *BMC Research Notes*.

[19] Engel ML, Holmes-Davis R, McCormick S (2005). Green sperm. Identification of male gamete promoters in *Arabidopsis*. *Plant Physiology*.

[20] Li J, Farmer AD, Lindquist IE (2012). Characterization of a set of novel meiotically-active promoters in *Arabidopsis*. *BMC Plant Biology*.

[21] Craigon DJ, James N, Okyere J, Higgins J, Jotham J, May S (2004). NASCArrays: a repository for microarray data generated by NASC's transcriptomics service. *Nucleic Acids Research*.

[22] Thomas-Chollier M, Defrance M, Medina-Rivera A (2011). RSAT 2011: regulatory sequence analysis tools. *Nucleic Acids Research*.

[23] Gubler F, Raventos D, Keys M, Watts R, Mundy J, Jacobsen JV (1999). Target genes and regulatory domains of the GAMYB transcriptional activator in cereal aleurone. *Plant Journal*.

[24] Higo K, Ugawa Y, Iwamoto M, Korenaga T (1999). Plant *cis*-acting regulatory DNA elements (PLACE) database: 1999. *Nucleic Acids Research*.

[25] Zambelli F, Pesole G, Pavesi G (2009). Pscan: Finding over-represented transcription factor binding site motifs in sequences from co-regulated or co-expressed genes. *Nucleic Acids Research*.

[26] Liseron-Monfils C, Lewis T, Ashlock D (2013). Promzea: a pipeline for discovery of co-regulatory motifs in maize and other plant species and its application to the anthocyanin and phlobaphene biosynthetic pathways and the Maize Development Atlas. *BMC Plant Biology*.

[27] Mahony S, Benos PV (2007). STAMP: a web tool for exploring DNA-binding motif similarities. *Nucleic Acids Research*.

[28] Bülow L, Brill Y, Hehl R (2010). AthaMap-assisted transcription factor target gene identification in *Arabidopsis thaliana*. *Database*.

[29] Bülow L, Engelmann S, Schindler M, Hehl R (2009). AthaMap, integrating transcriptional and post-transcriptional data. *Nucleic Acids Research*.

[30] Galuschka C, Schindler M, Bülow L, Hehl R (2007). AthaMap web tools for the analysis and identification of co-regulated genes. *Nucleic Acids Research*.

[31] Ole Steffens N, Galuschka C, Schindler M, Bülow L, Hehl R (2004). AthaMap: an online resource for *in silico* transcription factor binding sites in the *Arabidopsis thaliana* genome. *Nucleic Acids Research*.

[32] Steffens NO, Galuschka C, Schindler M, Bülow L, Hehl R (2005). AthaMap web tools for database-assisted identification of combinatorial cis-regulatory elements and the display of highly conserved transcription factor binding sites in *Arabidopsis thaliana*. *Nucleic Acids Research*.

[33] Yanagisawa S (2000). Dof1 and Dof2 transcription factors are associated with expression of multiple genes involved in carbon metabolism in maize. *Plant Journal*.

[34] Yanagisawa S, Schmidt RJ (1999). Diversity and similarity among recognition sequences of Dof transcription factors. *Plant Journal*.

[35] Yanagisawa S, Sheen J (1998). Involvement of maize Dof zinc finger proteins in tissue-specific and light-regulated gene expression. *Plant Cell*.

[36] Mena M, Vicente-Carbajosa J, Schmidt RJ, Carbonero P (1998). An endosperm-specific DOF protein from barley, highly conserved in wheat, binds to and activates transcription from the prolamin-box of a native B-hordein promoter in barley endosperm. *Plant Journal*.

[37] Gowik U, Burscheidt J, Akyildiz M (2004). *Cis*-regulatory elements for mesophyll-specific gene expression in the C_4_ plant *Flaveria trinervia*, the promoter of the C_4_ phosphoenolpyruvate carboxylase gene. *Plant Cell*.

[38] Sakai H, Aoyama T, Oka A (2000). *Arabidopsis* ARR1 and ARR2 response regulators operate as transcriptional activators. *Plant Journal*.

[39] Shirsat A, Wilford N, Croy R, Boulter D (1989). Sequences responsible for the tissue specific promoter activity of a pea legumin gene in tobacco. *MGG Molecular & General Genetics*.

[40] Reyes JC, Muro-Pastor MI, Florencio FJ (2004). The GATA family of transcription factors in arabidopsis and rice. *Plant Physiology*.

[41] Lam E, Chua NH (1989). ASF-2: a factor that binds to the cauliflower mosaic virus 35S promoter and a conserved GATA motif in *Cab* promoters.. *The Plant cell*.

[42] Filichkin SA, Leonard JM, Monteros A, Liu P, Nonogaki H (2004). A novel endo-*β*-mannanase gene in tomato LeMAN5 is associated with anther and pollen development. *Plant Physiology*.

[43] Rogers HJ, Bate N, Combe J (2001). Functional analysis of *cis*-regulatory elements within the promoter of the tobacco late pollen gene *g*10. *Plant Molecular Biology*.

[44] Stålberg K, Ellerstöm M, Ezcurra I, Ablov S, Rask L (1996). Disruption of an overlapping E-box/ABRE motif abolished high transcription of the *napA* storage-protein promoter in transgenic *Brassica napus* seeds. *Planta*.

[45] Plesch G, Ehrhardt T, Mueller-Roeber B (2001). Involvement of TAAAG elements suggests a role for Dof transcription factors in guard cell-specific gene expression. *Plant Journal*.

[46] Villain P, Mache R, Zhou D (1996). The mechanism of GT element-mediated cell type-specific transcriptional control. *The Journal of Biological Chemistry*.

[47] Buchel AS, Brederode FT, Bol JF, Linthorst HJM (1999). Mutation of GT-1 binding sites in the *Pr*-1*A* promoter influences the level of inducible gene expression *in vivo*. *Plant Molecular Biology*.

[48] Abe H, Urao T, Ito T, Seki M, Shinozaki K, Yamaguchi-Shinozaki K (2003). Arabidopsis AtMYC2 (bHLH) and AtMYB2 (MYB) function as transcriptional activators in abscisic acid signaling. *Plant Cell*.

[49] Chinnusamy V, Ohta M, Kanrar S (2003). ICE1: a regulator of cold-induced transcriptome and freezing tolerance in *Arabidopsis*. *Genes and Development*.

[50] Hartmann U, Sagasser M, Mehrtens F, Stracke R, Weisshaar B (2005). Differential combinatorial interactions of *cis*-acting elements recognized by R2R3-MYB, BZIP, and BHLH factors control light-responsive and tissue-specific activation of phenylpropanoid biosynthesis genes. *Plant Molecular Biology*.

[51] Eulgem T, Rushton PJ, Schmelzer E, Hahlbrock K, Somssich IE (1999). Early nuclear events in plant defence signalling: Rapid gene activation by WRKY transcription factors. *The EMBO Journal*.

[52] Zhang ZL, Xie Z, Zou X, Casaretto J, Ho TD, Shen QJ (2004). A rice *WRKY* gene encodes a transcriptional repressor of the gibberellin signaling pathway in aleurone cells. *Plant Physiology*.

[53] Simpson SD, Nakashima K, Narusaka Y, Seki M, Shinozaki K, Yamaguchi-Shinozaki K (2003). Two different novel *cis*-acting elements of *erd1*, a *clpA* homologous *Arabidopsis* gene function in induction by dehydration stress and dark-induced senescence. *Plant Journal*.

[54] Bovy A, Van Den Berg C, De Vrieze G, Thompson WF, Weisbeek P, Smeekens S (1995). Light-regulated expression of the *Arabidopsis thaliana* ferredoxin gene requires sequences upstream and downstream of the transcription initiation site. *Plant Molecular Biology*.

[55] Nakamura M, Tsunoda T, Obokata J (2002). Photosynthesis nuclear genes generally lack TATA-boxes: a tobacco photosystem I gene responds to light through an initiator. *Plant Journal*.

[56] Kucho K, Yoshioka S, Taniguchi F, Ohyama K, Fukuzawa H (2003). *Cis*-acting elements and DNA-binding proteins involved in CO_2_-responsive transcriptional activation of *Cah1* encoding a periplasmic carbonic anhydrase in *Chlamydomonas reinhardtii*. *Plant Physiology*.

[57] Yoshioka S, Taniguchi F, Miura K, Inoue T, Yamano T, Fukuzawa H (2004). The novel Myb transcription factor LCR1 regulates the CO_2_-responsive gene *Cah1*, encoding a periplasmic carbonic anhydrase in *Chlamydomonas reinhardtii*. *Plant Cell*.

[58] Hirayama T, Shinozaki K (1996). A cdc5+ homolog of a higher plant, *Arabidopsis thaliana*. *Proceedings of the National Academy of Sciences of the United States of America*.

[59] Lin Z, Yin K, Zhu D, Chen Z, Gu H, Qu L (2007). AtCDC5 regulates the G2 to M transition of the cell cycle and is critical for the function of Arabidopsis shoot apical meristem. *Cell Research*.

[60] Liang X, Nazarenus TJ, Stone JM (2008). Identification of a consensus DNA-binding site for the *Arabidopsis thaliana* SBP domain transcription factor, AtSPL14, and binding kinetics by surface plasmon resonance. *Biochemistry*.

[61] Kagaya Y, Ohmiya K, Hattori T (1999). RAV1, a novel DNA-binding protein, binds to bipartite recognition sequence through two distinct DNA-binding domains uniquely found in higher plants. *Nucleic Acids Research*.

[62] Hu YX, Wang YH, Liu XF, Li JY (2004). Arabidopsis RAV1 is down-regulated by brassinosteroid and may act as a negative regulator during plant development. *Cell Research*.

[63] Sohn KH, Lee SC, Jung HW, Hong JK, Hwang BK (2006). Expression and functional roles of the pepper pathogen-induced transcription factor RAV1 in bacterial disease resistance, and drought and salt stress tolerance. *Plant Molecular Biology*.

[64] Yamasaki K, Kigawa T, Inoue M (2004). Solution structure of the B3 DNA binding domain of the Arabidopsis cold-responsive transcription factor RAV1. *Plant Cell*.

[65] Woo HR, Kim JH, Lee U (2010). The RAV1 transcription factor positively regulates leaf senescence in *Arabidopsis*. *Journal of Experimental Botany*.

[66] Berrocal-Lobo M, Molina A (2004). Ethylene response factor 1 mediates *Arabidopsis* resistance to the soilborne fungus *Fusarium oxysporum*. *Molecular Plant-Microbe Interactions*.

[67] Berrocal-Lobo M, Molina A, Solano R (2002). Constitutive expression of *ETHYLENE - RESPONSE - FACTOR1* in *Arabidopsis* confers resistance to several necrotrophic fungi. *Plant Journal*.

[68] Hao D, Ohme-Takagi M, Sarai A (1998). Unique mode of GCC box recognition by the DNA-binding domain of ethylene-responsive element-binding factor (ERF domain) in plant. *Journal of Biological Chemistry*.

[69] Ohme-Takagi M, Shinshi H (1995). Ethylene-inducible DNA binding proteins that interact with an ethylene-responsive element. *Plant Cell*.

[70] Solano R, Stepanova A, Chao Q, Ecker JR (1998). Nuclear events in ethylene signaling: A transcriptional cascade mediated by ETHYLENE-INSENSITIVE3 and ETHYLENE-RESPONSE-FACTOR1. *Genes and Development*.

[71] Niu X, Helentjaris T, Bate NJ (2002). Maize ABI4 binds coupling element1 in abscisic acid and sugar response genes. *Plant Cell*.

[72] Mathieu J, Yant LJ, Mürdter F, Küttner F, Schmid M (2009). Repression of flowering by the miR172 target SMZ. *PLoS Biology*.

[73] Kharazmi J, Moshfegh C (2013). Investigation of *dmyc* promoter and regulatory regions. *Gene Regulation and Systems Biology*.

[74] Grace ML, Chandrasekharan MB, Hall TC, Crowe AJ (2004). Sequence and spacing of TATA box elements are critical for accurate initiation from the *β*-phaseolin promoter. *The Journal of Biological Chemistry*.

[75] Creux NM, Ranik M, Berger DK, Myburg AA (2008). Comparative analysis of orthologous cellulose synthase promoters from *Arabidopsis*, *Populus* and *Eucalyptus*: evidence of conserved regulatory elements in angiosperms. *New Phytologist*.

[76] Helin K (1998). Regulation of cell proliferation by the E2F transcription factors. *Current Opinion in Genetics and Development*.

[77] Yu F, Huaxia Y, Lu W, Wu C, Cao X, Guo X (2012). *GhWRKY15*, a member of the WRKY transcription factor family identified from cotton (*Gossypium hirsutum* L.), is involved in disease resistance and plant development. *BMC Plant Biology*.

[78] Hsieh C (1994). Dependence of transcriptional repression on CpG methylation density. *Molecular and Cellular Biology*.

[79] López Y, Patil A, Nakai K (2013). Identification of novel motif patterns to decipher the promoter architecture of co-expressed genes in *Arabidopsis thaliana*. *BMC Systems Biology*.

[80] Hattori T, Terada T, Hamasuna S (1995). Regulation of the *Osem* gene by abscisic acid and the transcriptional activator VP1: analysis of *cis*-acting promoter elements required for regulation by abscisic acid and VP1. *Plant Journal*.

[81] Lacombe E, van Doorsselaere J, Boerjan W, Boudet AM, Grima-Pettenati J (2000). Characterization of *cis*-elements required for vascular expression of the *Cinnamoyl CoA Reductase* gene and for protein—DNA complex formation. *The Plant Journal*.

[82] Loppes R, Radoux M (2001). Identification of short promoter regions involved in the transcriptional expression of the nitrate reductase gene in *Chlamydomonas reinhardtii*. *Plant Molecular Biology*.

[83] Jiao Y, Meyerowitz EM (2010). Cell-type specific analysis of translating RNAs in developing flowers reveals new levels of control. *Molecular Systems Biology*.

[84] Zhao Y, Medrano L, Ohashi K (2004). Hanaba taranu is a GATA transcription factor that regulates shoot apical meristem and flower development in *Arabidopsis*. *Plant Cell*.

[85] Mara CD, Irish VF (2008). Two GATA transcription factors are downstream effectors of floral homeotic gene action in *Arabidopsis*. *Plant Physiology*.

[86] de Veylder L, Beeckman T, Inzé D (2007). The ins and outs of the plant cell cycle. *Nature Reviews Molecular Cell Biology*.

[87] Lammens T, Li J, Leone G, De Veylder L (2009). Atypical E2Fs: new players in the E2F transcription factor family. *Trends in Cell Biology*.

[88] Godoy M, Franco-Zorrilla JM, Pérez-Pérez J, Oliveros JC, Lorenzo Ó, Solano R (2011). Improved protein-binding microarrays for the identification of DNA-binding specificities of transcription factors. *Plant Journal*.

[89] Jain M, Tyagi AK, Khurana JP (2008). Constitutive expression of a meiotic recombination protein gene homolog, *OsTOP6A1*, from rice confers abiotic stress tolerance in transgenic *Arabidopsis* plants. *Plant Cell Reports*.

[90] Lu BC (1974). Genetic recombination in *Coprinus*. IV. A kinetic study of the temperature effect on recombination frequency. *Genetics*.

[91] Lu BC (2000). The control of meiosis progression in the fungus *Coprinus cinereus* by light/dark cycles. *Fungal Genetics and Biology*.

[92] Niu X, Renshaw-Gegg L, Miller L, Guiltinan MJ (1999). Bipartite determinants of DNA-binding specificity of plant basic leucine zipper proteins. *Plant Molecular Biology*.

