# Supplementary material for: Characterization of Putative cis-Regulatory Elements in Genes Preferentially Expressed in Arabidopsis Male Meiocytes
Source: Biomed Res Int. 2014 Aug 27;2014:708364. doi: 10.1155/2014/708364 (PMC4163388; doi:10.1155/2014/708364)
Supplement: Supplementary file 1 — Supplemental File 1 presents the difference in expression between meiocytes and anther, the difference in expression between meiocytes and seedlings, the annotated function, and the GO functional categorization of the 50 candidate genes used for promoter study. Supplemental File 2 lists the 50 randomly selected genes used as a negative control. Supplemental File 3 shows the descriptions of the overrepresented sequence in the promoters of 50 randomly selected genes, detected using Pscan. Supplemental File 4 shows the sequence logos of novel motifs in the promoters of 50 genes preferentially expressed during meiosis and 50 randomly selected genes, detected using Promzea. Supplemental File 5 shows the sequence logos of novel motifs in the promoters of 50 randomly selected genes, detected using Promzea, and the best match of each motif in the PLACE database. [file 708364.f1.zip › 1031476.doc]

**An statistically overrepresented motif detected by Pscan in promoters of 50 randomly selected genes.**

Table. A statistically overrepresented motif in promoters of 50 randomly selected genes, detected by Pscan.

| **Motif ID** | **TF_Name** | **Class of the TF** | **Family of the TF** | ***p*-value** | **Reference** |
| --- | --- | --- | --- | --- | --- |
| MA0120.1 | ID1 | Zinc-coordinating | BetaBetaAlpha-zinc finger | 0.00705542 |  |

Figure. Sequence logo of the motif MA0120.1 detected by Pscan. Letters in the logos abbreviate the nucleotides (A, C, G, T) and are sized relative to their occurrence.

**Reference**

1. Kozaki A, Hake S, Colasanti J (2004) The maize ID1 flowering time regulator is a zinc finger protein with novel DNA binding properties. Nucleic Acids Res 32: 1710-1720.
