# Supplementary material for: Characterization of Putative cis-Regulatory Elements in Genes Preferentially Expressed in Arabidopsis Male Meiocytes
Source: Biomed Res Int. 2014 Aug 27;2014:708364. doi: 10.1155/2014/708364 (PMC4163388; doi:10.1155/2014/708364)
Supplement: Supplementary file 1 — Supplemental File 1 presents the difference in expression between meiocytes and anther, the difference in expression between meiocytes and seedlings, the annotated function, and the GO functional categorization of the 50 candidate genes used for promoter study. Supplemental File 2 lists the 50 randomly selected genes used as a negative control. Supplemental File 3 shows the descriptions of the overrepresented sequence in the promoters of 50 randomly selected genes, detected using Pscan. Supplemental File 4 shows the sequence logos of novel motifs in the promoters of 50 genes preferentially expressed during meiosis and 50 randomly selected genes, detected using Promzea. Supplemental File 5 shows the sequence logos of novel motifs in the promoters of 50 randomly selected genes, detected using Promzea, and the best match of each motif in the PLACE database. [file 708364.f1.zip › 1031477.doc]

Figure. Sequence logos of novel motifs in the promoters of 50 genes preferentially expressed during meiosis (left panel) and 50 randomly selected genes (right panel), detected using Promzea. Letters in the logos abbreviate the nucleotides (A, C, G, T) and are sized relative to their occurrence. Each predicted motif is provided with a Mean Normalized Conditional Probability (MNCP); a MNCP score greater than 1 indicates that the motif is more represented in the input data set compared to a random set of promoters/first introns.
